# Supplementary material for: A metal-free photoactive nitrogen-doped carbon nanosolenoid with broad absorption in visible region for efficient photocatalysis
Source: Nat Commun. 2023 Sep 20;14:5831. doi: 10.1038/s41467-023-41467-4 (PMC10511729; doi:10.1038/s41467-023-41467-4)
Supplement: Supplementary file 1 — Supplementary Information [file 41467_2023_41467_MOESM1_ESM.pdf]

Supplementary Information

A metal-free photoactive nitrogen-doped carbon nanosolenoid with broad absorption in visible region for efficient photocatalysis

Yu Zhou,[†1,2] Xinyu Zhang,[†2] Guan Sheng,[†3] Shengda Wang,[2] Muqing Chen,*[1] Guilin Zhuang,[4] Yihan Zhu,*[3] Pingwu Du*[2]

[1] School of Materials Science and Engineering, Dongguan University of Technology, Dongguan, Guangdong Province, 523808, China

[2] Key Laboratory of Precision and Intelligent Chemistry, Anhui Laboratory of Advanced Photon Science and Technology, Department of Materials Science and Engineering, University of Science and Technology of China, 96 Jinzhai Road, Hefei, Anhui Province, 230026, China

[3] Center for Electron Microscopy, Institute for Frontier and Interdisciplinary Sciences, State Key Laboratory Breeding Base of Green Chemistry Synthesis Technology, College of Chemical Engineering, Zhejiang University of Technology, 18 Chaowang Road, Hangzhou 310014, Zhejiang Province, 310014, China

[4] College of Chemical Engineering, Zhejiang University of Technology, 18 Chaowang Road, Hangzhou, Zhejiang Province, 310014, China

[†] These authors contributed equally: Yu Zhou, Xinyu Zhang, Guan Shen

*Corresponding author: *Corresponding author: mqchen@ustc.edu.cn; yihanzhu@zjut.edu.cn; dupingwu@ustc.edu.cn

Tel/Fax: 86-551-63606207

Synthetic Details

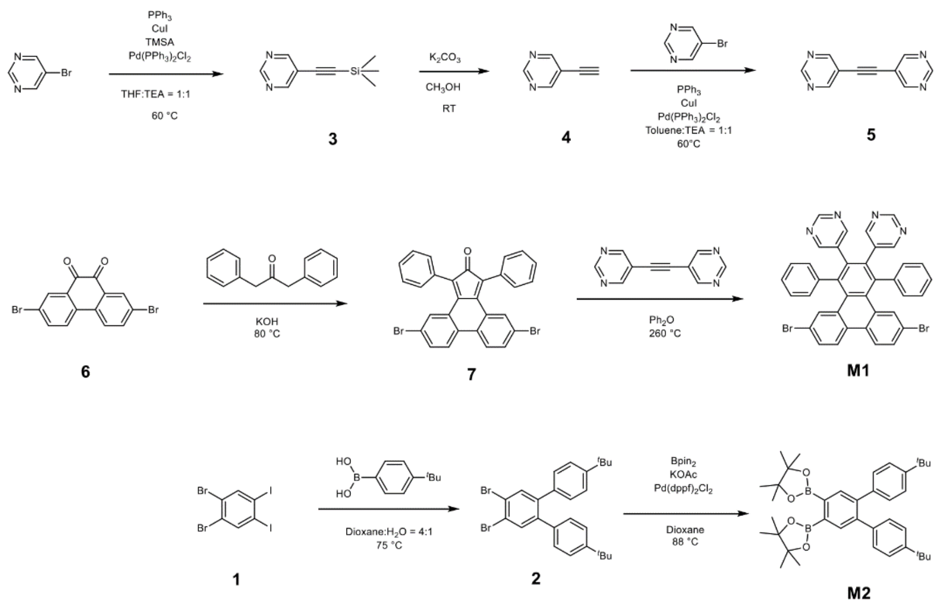

Figure S1. Synthesis procedure of compounds **M1** and **M2**.

Compound **3-7** and **M1** were prepared according to the published procedures [S1-S3].

Synthesis of compound 4. A mixture of tetrahydrofuran (20 mL), triethylamine (20 mL), 5-bromopyrimidine (5 g, 31.5 mmol), triphenylphosphine (0.366 g, 1.39 mmol), copper (I) idodide (1.06 g, 5.56 mmol), Bis(triphenylphosphine)palladium(II) dichloride was degased for 15 minutes with nitrogen gas bubbling through the system and ethynyl(trimethyl)silane was added to the reaction flask while degassing. The reaction mixture was heated to 60 ℃ for 15 hours, then cooled down to room temperature, concentrated to dryness under reduced pressure and the resulted solid was washed with diethyl ether (2×50 mL) and dried under vacuum to get crude product compound **3** without further purification. Methanol (30 mL) and potassium carbonate (7.68 g, 55.6 mmol) were added to compound **3** and the reaction mixture was agitated

at room temperature for 2 hours. The reaction mixture was concentrated to dryness under reduced pressure and the resulted solid was washed with ethyl acetate (2×50 mL) and dried under reduced pressure to obtain crude product compound **4** (3 g, 93% yield) and used without additional purification. ^{1}H NMR ($CDCl_3$, 400 MHz): δ 9.12 (s, 1H), 8.83 (s, 2H), 3.40 (s, 1H).

Synthesis of compound 5. A mixture of toluene (20 mL), triethylamine (20 mL), triphenyl phosphine (100 mg, 0.38 mmol), copper (I) iodide (146 mg, 0.77 mmol), 5-bromopyrimidine (3.2 g, 20.1 mmol) was degased for 15 minutes. Bis(triphenylphosphine)palladium(II) dichloride (539 mg, 0.77 mmol) was added to the degased reaction mixture and the mixture was degassed for another 10 minutes. The reaction was heated to 60 ℃ and then compound **4** (2.0 g, 20.0 mmol) was added. The reaction mixture was stirred at 60 ℃ for 18 hours, filtered and washed with ethyl acetate. The filtrate was dried and purified using column chromatography with ethyl acetate and methanol (95:5) yielding 2.9 g (82%) of the product. ^{1}H NMR ($CDCl_3$, 400 MHz): δ 9.23 (s, 2H), 8.92 (s, 4H).

Synthesis of compound 6. Phenanthrene-9,10-dione (3.5 g, 16.8 mmol) was dissolved in 98% H_2SO_4 (190 mL) and N-Bromosuccinimide (6.4 g, 36.0 mmol) was added in portions over a period of 40 minutes with continuous stirring. The mixture was vigorously stirred at room temperature for 3 days, then the reaction mixture was poured into a large beaker containing crushed ice and left for 1 hour with stirring. The reaction product was filtered and washed thoroughly with water. The crude product was washed by DMSO to afford compound **6** as orange solid (5.0 g, 81%). ^{1}H NMR (DMSO-d_6,

400 MHz): δ 8.23 (d, 2H), 8.05 (d, 2H), 7.93 (d, 2H).

Synthesis of compound 7. 1,3-diphenylpropanone (1.72 g, 8.2 mmol) and **6** (2.46 g, 6.7 mmol) were added to methanol (15 mL) under stirring. The reaction mixture was heated to 85 ℃ and a solution of potassium hydroxide (305 mg, 5.5 mmol) in methanol (1.5 mL) was added dropwise. The reaction mixture was maintaining at the same temperature with vigorous stirring for 15 minutes. The cold solution was then filtered and washed with methanol to afford compound **7** as a gray-green solid (3.1 g, 85%). ^{1}H NMR ($CDCl_3$, 400 MHz): δ 7.62 (s, 2H), 7.60 (s, 2H), 7.50-7.41 (m, 8H), 7.38 (d, 4H).

Synthesis of compound 5,5'-(6,11-dibromo-1,4-diphenyltriphenylene-2,3-diyl)dipyrimidine (M1). In a reaction flask, **7** (0.855 g, 1.47 mmol) , **5** (0.275 g, 1.51 mmol) and 3 mL of diphenyl ether were heated to reflux for 2 hours (NOTE: heating for more than 2 h leads to decomposition of the product). The reaction mixture was cooled to ambient temperature and purified using column chromatography with hexane and ethyl acetate (1:1) then 100% ethyl acetate to afford compound **M1** (0.723 g, 69%). ^{1}H NMR ($CDCl_3$, 400 MHz): δ 8.87 (s, 2H), 8.25 (s, 2H), 8.11 (s, 4H), 7.72 (d, 2H), 7.57 (d, 2H), 7.28 (m, 6H), 7.05 (m, 4H).

Synthesis of compound 1. Compound **1** and **2** were prepared according to the published procedures[S4-S6]. An oven dried 250 mL round bottom flask containing a fine grounded iodine (3.43 g, 13.5 mmol), $NaIO_3$ (1.34 g, 6.76 mmol). Sulfuric acid (98%, 150 mL) was slowly added to the flask and the mixture was stirred at 40 ℃ for 1 hour in the dark (the flask was shielded by aluminum foil during the entire reaction process).

The resulting solution was then cooled to 0 ℃ and 1,2-dibromobenzene (4.00 g, 16.9 mmol) was added in one portion. The mixture was vigorously stirred at 0 ℃ for 4 hours, and then was poured into ice water. The resulting white precipitate was filtered off, washed with much water, dissolved in $CHCl_3$ and washed with 10% $NaHSO_3$ (aq.), dried over anhydrous $MgSO_4$, and then concentrated by rotary evaporator. The crude product was purified by crystallization from $CHCl_3$/hexanes to afforded compound **1** (6.7 g, 81%) as a white crystalline solid. ^{1}H NMR ($CDCl_3$, 400 MHz): δ 8.05 (s, 2H).

Synthesis of compound 2. To a degassed suspension of **1** (2 g, 5.32 mmol), (4-tert-butylphenyl)boronic acid (1.89 g, 10.64 mmol), $Ba(OH)_2.8H_2O$ (6.72 g, 21.29 mmol), n-Bu_4NBr (172 mg, 0.53 mmol), and $Pd(PPh_3)_4$ (184.42 mg, 0.16 mmol) were added in dioxane/H_2O (52 mL : 13 mL). The mixture was bubbled with argon for 15 minutes, then the flask was sealed and heated to 80 °C for 24 hours. After reaction, 1 M HCl (40 mL) was added and most of dioxane was removed by rotary evaporation, the product was extracted with CH_2Cl_2 and washed twice with brine, dried over anhydrous $MgSO_4$ and concentrated under rotary evaporation. The crude product was passed through a silica gel column chromatography with petroleum ether as the eluent, and recrystallization from CH_2Cl_2/MeOH (v/v, 1:10) to afford compound **2** as white solid (1.8 g, 54%). ^{1}H NMR ($CDCl_3$, 400 MHz): δ 7.66 (s, 2H), 7.22 (d, J = 8.4 Hz, 4H), 7.01 (d, J = 8.8 Hz, 4H), 1.28 (s, 18H).

Synthesis of compound 2,2'-(4,4''-di-*tert*-butyl-[1,1':2',1''-terphenyl]-4',5'-diyl)bis(4,4,5,5-tetramethyl-1,3,2-dioxaborolane) (M2). A 250 mL flask containing **2** (3 g, 4.64 mmol), $Pd(dppf)Cl_2$ (383 mg, 524 μmol), dried KOAc (2.27 g, 23.2 mmol),

and bis(pinacolato)diboron (4.71 g, 18.56 mmol) was evacuated and filled with argon three times. Dry 1,4-dioxane (60 mL) was transferred to the flask via syringe under nitrogen at room temperature. The mixture was then stirred at 100 °C for 48 h. The residue was purified by silica gel column chromatography (CH_2Cl_2/petroleum ether = 1/2) to give **M2** as a white solid (3 g, 55%). ^1H NMR ($CDCl_3$, 400 MHz): δ 7.70 (s, 2H), 7.20 (d, J = 8.5 Hz, 4H), 7.07 (d, J = 8.5 Hz, 4H), 1.36 (s, 24H), 1.28 (s, 18H); ^{13}C NMR ($CDCl_3$, 100 MHz): δ 149.15 (s), 141.07 (s), 138.66 (s), 135.97 (s), 129.49 (s), 124.53 (s), 83.85 (s), 34.38 (s), 31.34 (s), 24.92 (s); HR-MS (MALDI-TOF) *m/z* calcd. for $C_{32}H_{36}B_2O_4$ $[M]^+$: 594.4052, found:594.4013.

General Procedure for Photoredox Reactions.

All the products are known and ^1H NMR spectra matches with previously reported data[S7-12].

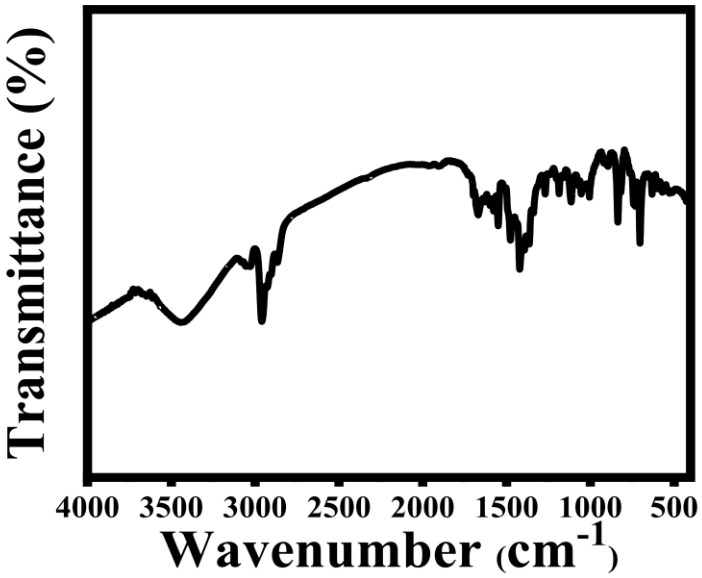

Figure S2. FTIR spectrum of **P1**.

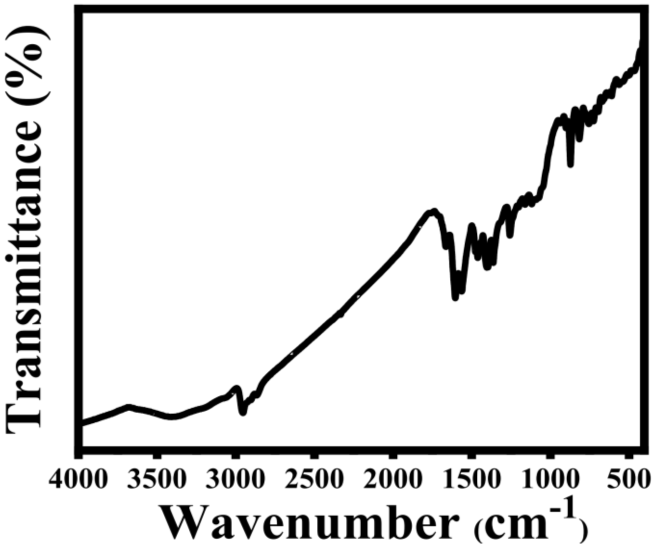

Figure S3. FTIR spectrum of **N-CNS**.

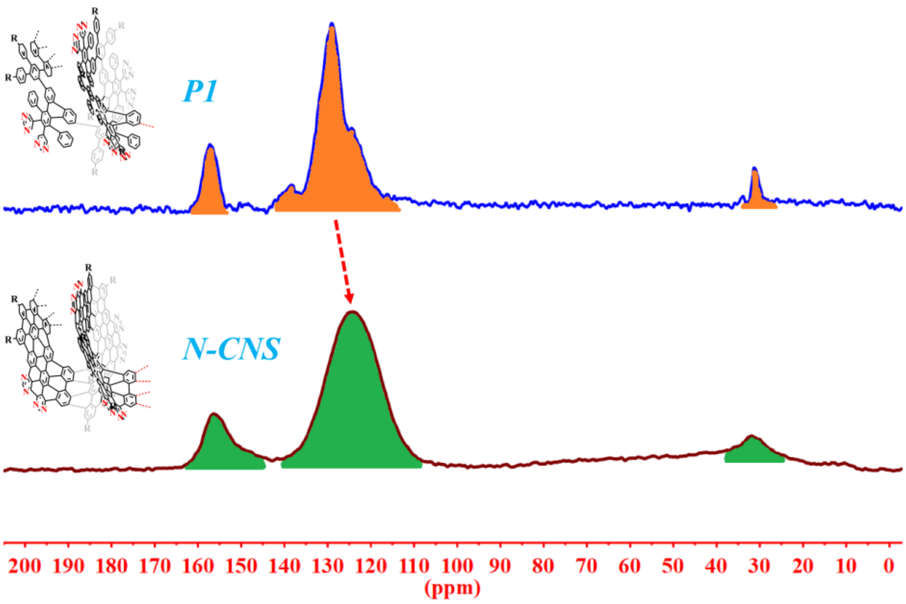

Figure S4. Solid-state ^{13}C NMR spectra of **P1** and **N-CNS**.

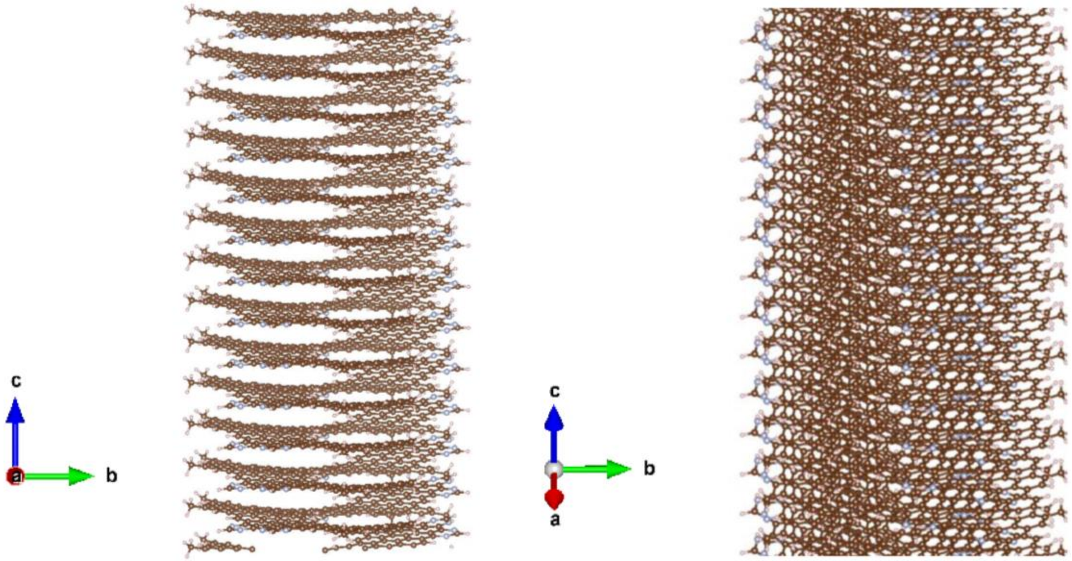

Figure S5. [100] and [201] projections of the proposed 1D periodic structural model of **N-CNS**.

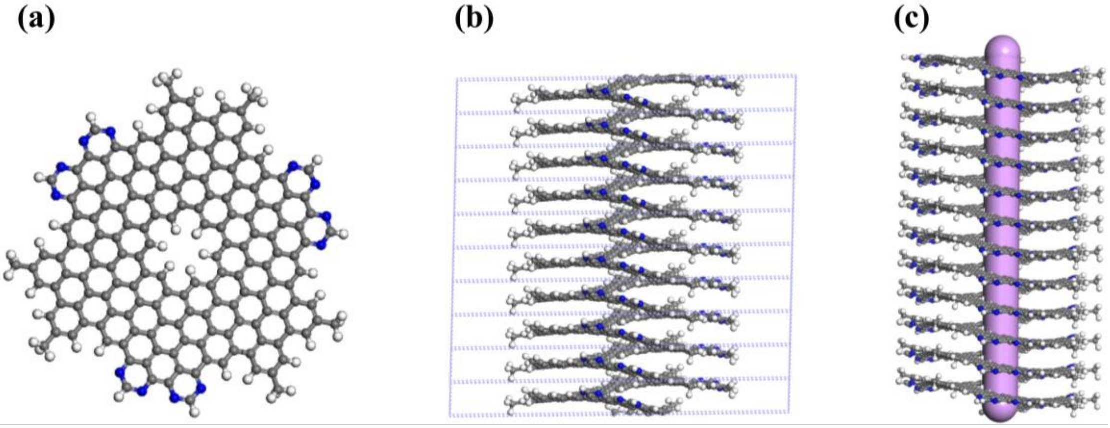

Figure S6. Relaxed structure of **N-CNS** with (a) top view, (b) side view and (c) diagrammatic sketch.

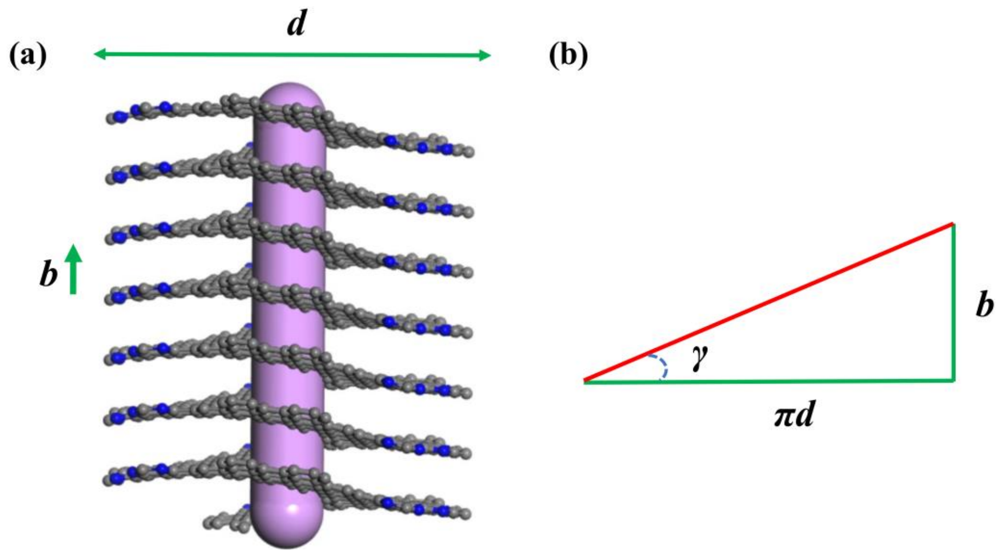

Figure S7. (a) Helical structure of **N-CNS** with the removal of substituent group; (b) the scheme of lead angle.

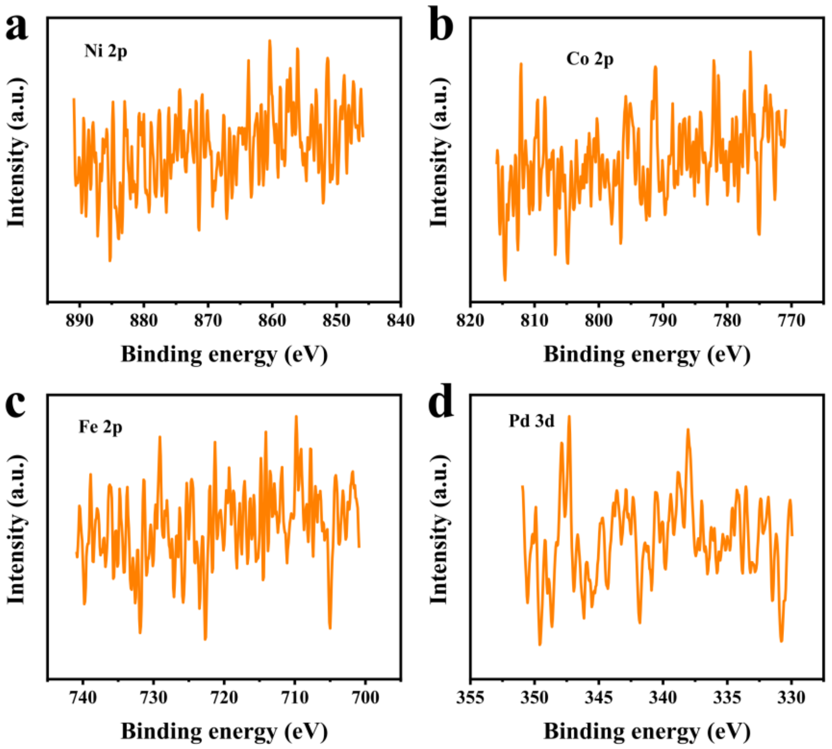

Figure S8. (a) Ni 2p, (b) Co 2p, (c) Fe 2p, (d) Pd 3d XPS spectra of **N-CNS**.

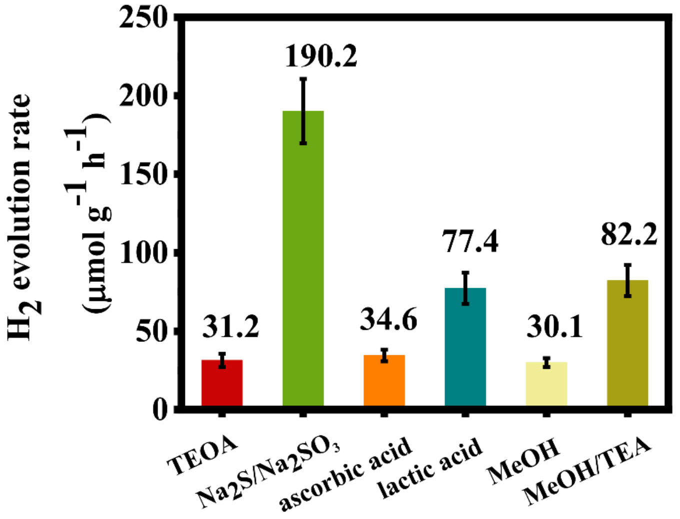

Figure S9. H_2 evolution rates of **N-CNS** photocatalyst using different sacrificial electron donors (red: 20 vol% TEOA; green: 0.75 M Na_2S and 1.05 M Na_2SO_3; orange: 0.5 M ascorbic acid; dark cyan: 10 vol% lactic acid; yellow: 20 vol% MeOH; dark yellow: 20 vol% MeOH and 20 vol% TEA). The error bars show standard deviation (SD).

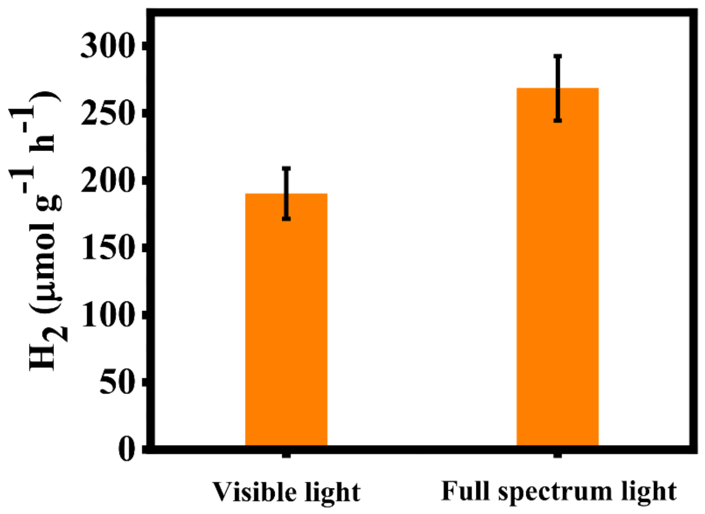

Figure S10. H_2 evolution rates under visible light and full spectrum light irradiation.

The error bars show standard deviation (SD).

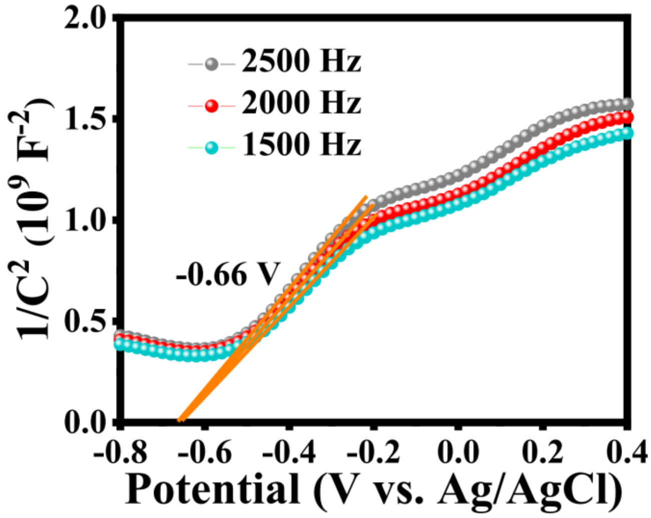

Figure S11. Mott-Schottky plot of **N-CNS**.

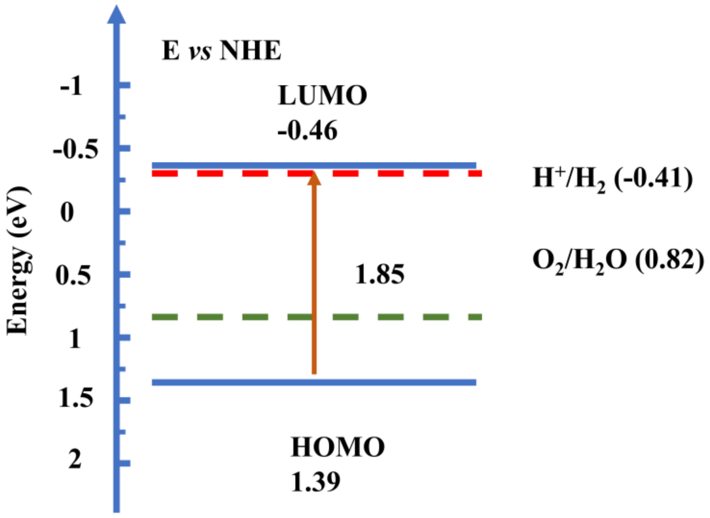

Figure S12. Band-structure diagram for **N-CNS**.

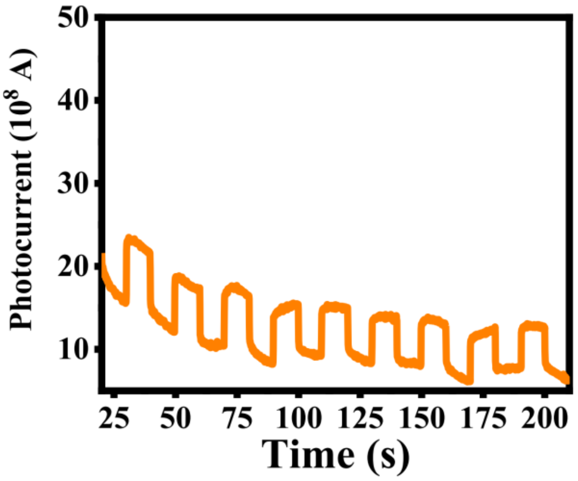

Figure S13. Transient photocurrent response of **N-CNS** at 1.23 V vs. RHE.

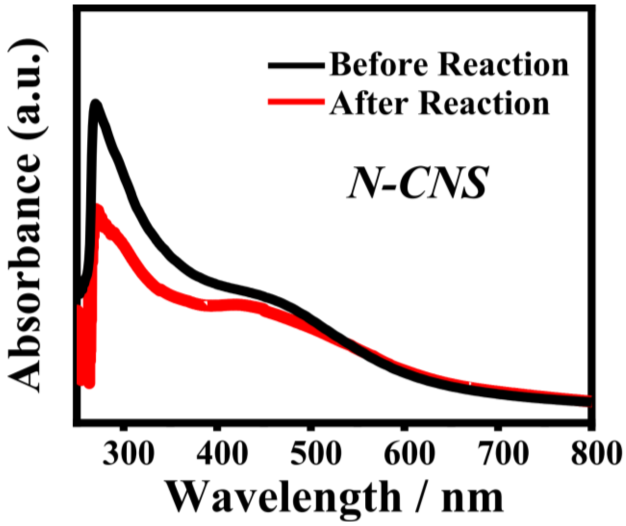

Figure S14. The absorption spectra of **N-CNS** before and after photocatalytic aza-Henry reaction.

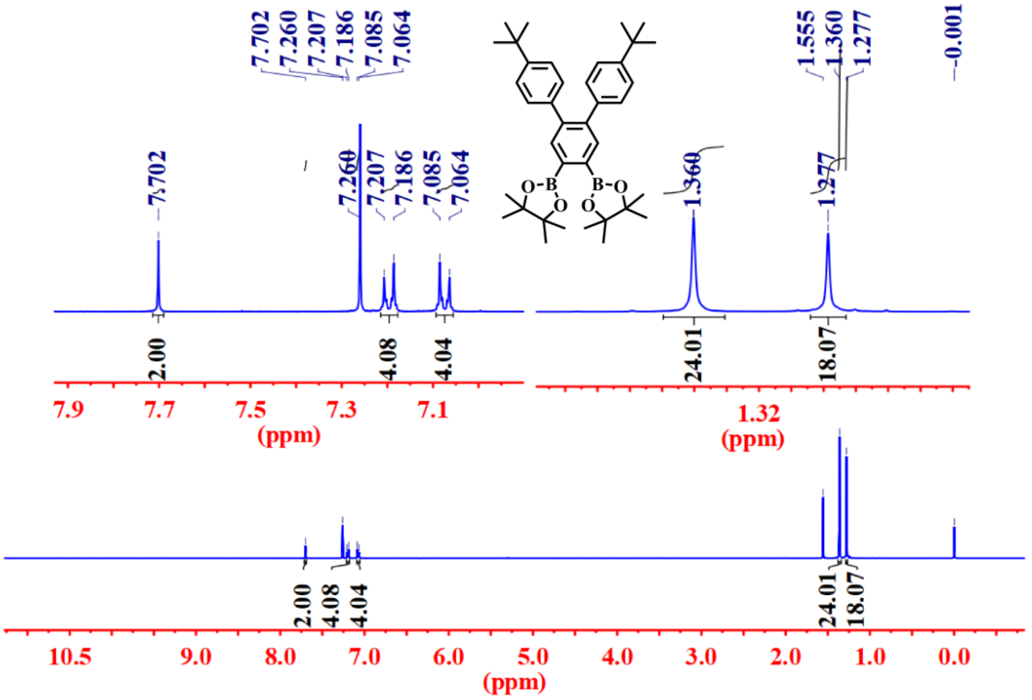

Figure S15. ^{1}H NMR spectrum of compound **M2** in $CDCl_3$.

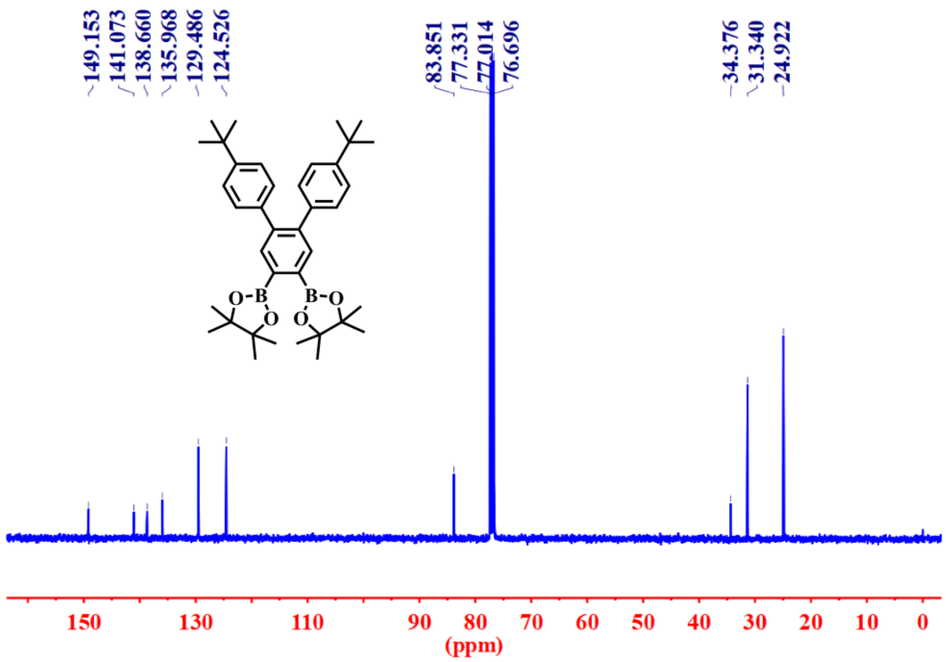

Figure S16. ^{13}C NMR spectrum of compound **M2** in $CDCl_3$.

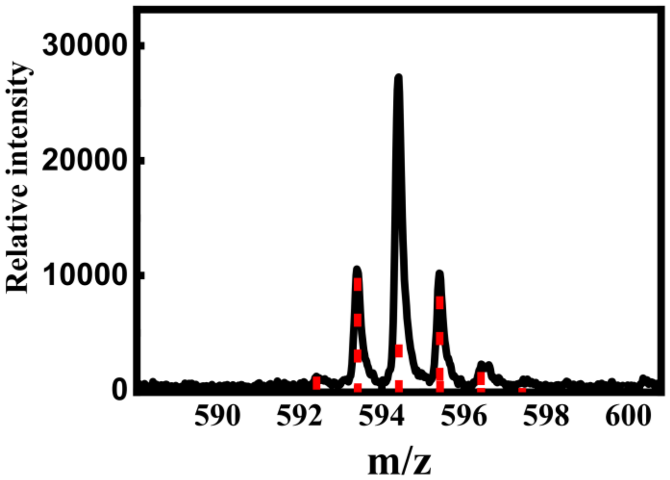

Figure S17. MALDI-TOF-MS spectrum (black) and simulated data (red) for compound **M2**.

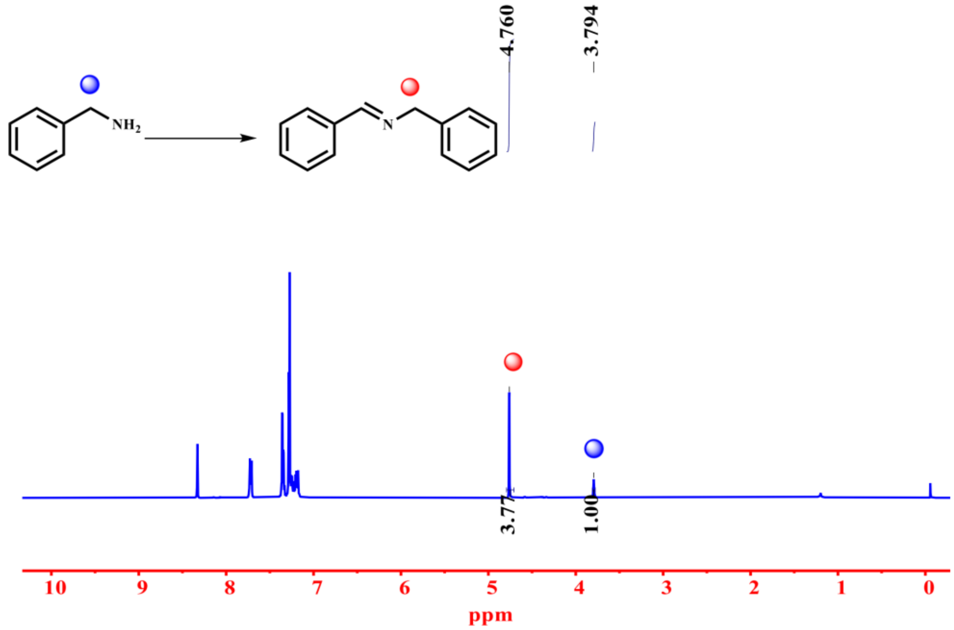

Figure S18. ^{1}H NMR spectrum of products from the photocatalytic homocoupling of benzylamine ($CDCl_3$).

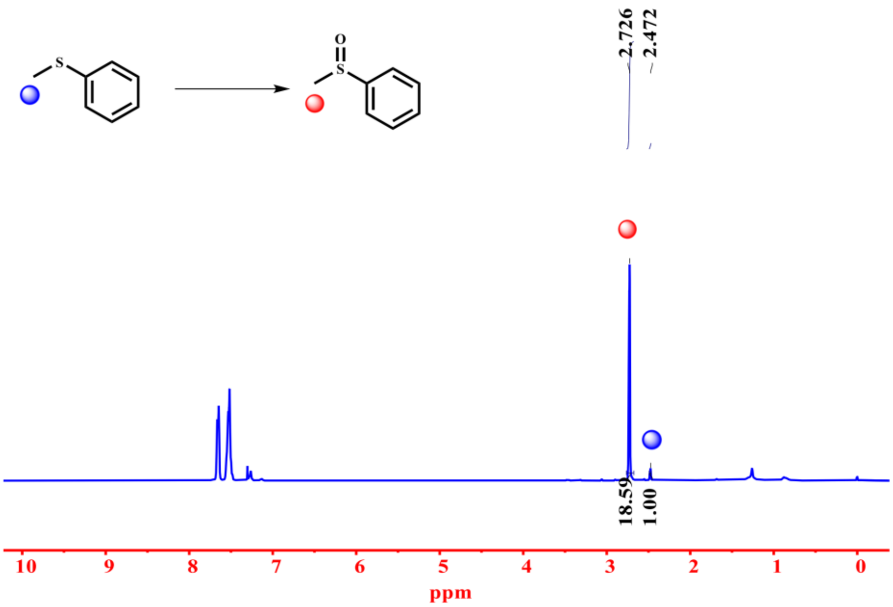

Figure S19. ^{1}H NMR spectrum of products from the photocatalytic thioanisole oxidation ($CDCl_3$).

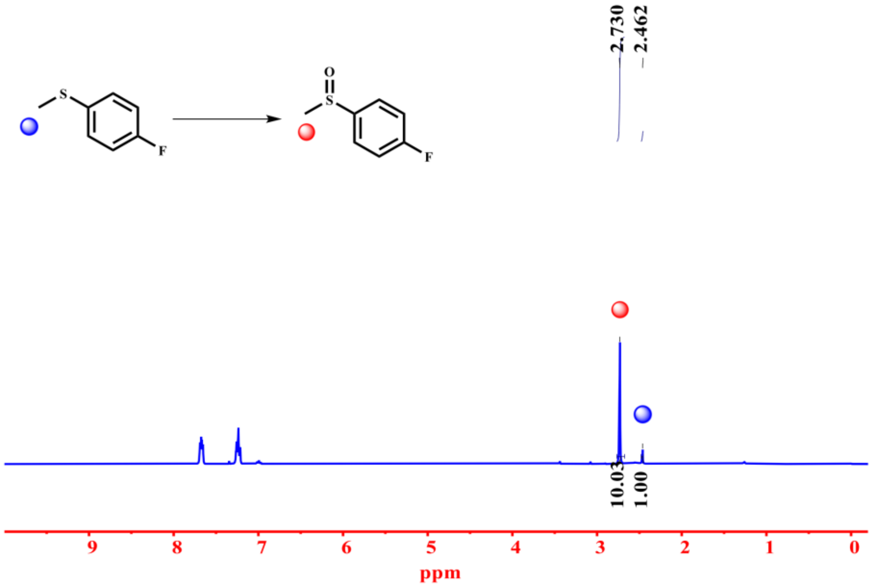

Figure S20. ^{1}H NMR spectrum of products from the photocatalytic (4-fluorophenyl)(methyl)sulfane oxidation ($CDCl_3$).

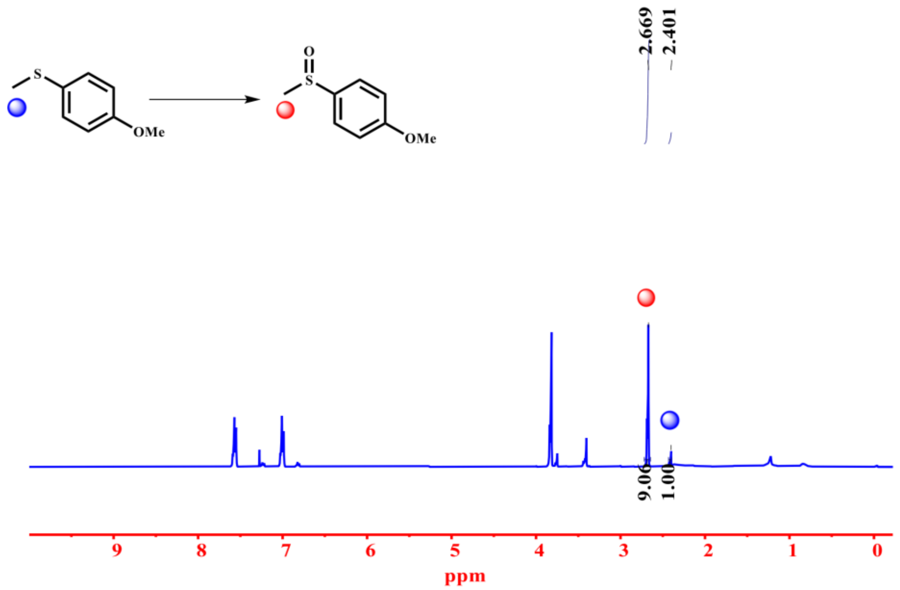

Figure S21. ^{1}H NMR spectrum of products from the photocatalytic (4-methoxyphenyl)(methyl)sulfane oxidation ($CDCl_3$).

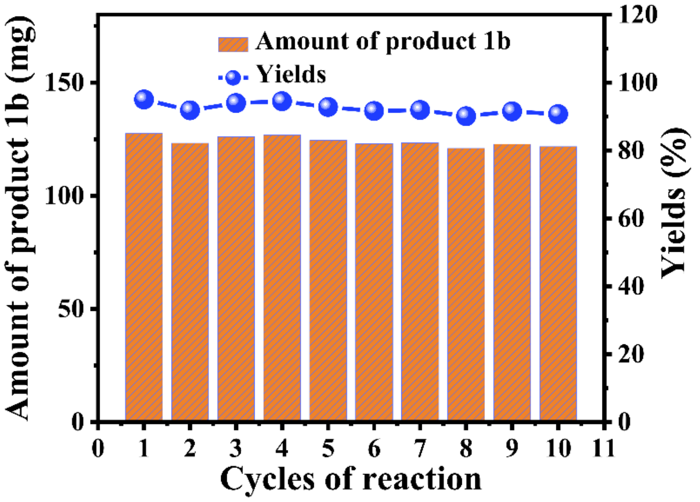

Figure S22. Cycle stability of photocatalytic aza-Henry reactions for **N-CNS** sample. Reaction conditions: 2-phenyl-1,2,3,4-tetrahydroisoquinoline **1a** (0.5 mmol, 104.6 mg), photocatalyst (1.5 mg), CH_3NO_2 (3 mL), O_2 (1 atm), LED lamp (9 W).

Table S1. Coordinate and lattice parameters of **N-CNS** polymers.

Coordinate of **N-CNS** unit cell with lattice parameters. (a= 41.4429 Å, b= 41.1762 Å, c= 4.10040Å, α= 90.1662°, β= 88.0819°, γ= 90.3638°)							
**C**	22.784593	23.679264	3.425172	C	13.223863	14.168563	2.202178
**C**	23.281209	22.380962	3.550111	C	11.258450	19.525277	1.800998
**C**	24.637340	22.112216	3.711187	C	9.883164	19.729432	1.642983
**C**	25.553155	23.197410	3.692807	C	8.984497	18.663991	1.586703
**C**	25.071160	24.529958	3.654400	C	9.474194	17.366586	1.719910
**C**	23.680749	24.777196	3.539723	C	10.839325	17.100687	1.885574
**C**	25.141112	20.762438	3.875945	C	23.736855	17.295716	0.484738
**C**	26.539630	20.525086	3.798046	C	22.398835	17.519673	0.804591
**C**	27.445310	21.618440	3.680134	C	21.514830	16.486449	1.098991
**C**	26.956318	22.951630	3.694426	C	21.988862	15.147598	1.046390
**C**	27.038409	19.198906	3.856445	C	23.352054	14.889503	0.746361
**C**	28.835385	21.376398	3.575893	C	24.236666	15.965107	0.479974
**C**	29.734559	22.468901	3.509684	C	20.126975	16.743510	1.431362
**C**	29.255410	23.788072	3.601763	C	19.235189	15.649468	1.595124
**C**	27.863438	24.037526	3.683788	C	19.723206	14.311168	1.548905
**C**	30.178518	24.873988	3.613064	C	21.098159	14.061226	1.291783
**C**	28.293495	26.469590	3.836201	C	19.634278	18.035693	1.598759
**C**	27.384371	25.379496	3.755188	C	18.286939	18.295454	1.844851
**C**	29.335814	20.040163	3.544095	C	17.365743	17.214267	1.875917
**C**	30.723942	19.793197	3.364088	C	17.852236	15.887323	1.800513
**C**	31.636885	20.918873	3.213860	C	18.837408	13.230363	1.777907
**C**	31.130260	22.237749	3.338786	C	19.335705	11.904428	1.791606
**C**	27.798358	27.764533	3.970278	C	20.693851	11.655298	1.524555
**C**	26.424358	28.031993	3.982340	C	21.579351	12.729926	1.258391
**C**	25.504083	26.958025	3.813953	C	21.172653	10.312153	1.520412
**C**	25.988641	25.626485	3.738209	C	22.524966	10.026408	1.205531
**C**	33.717466	21.792615	2.860729	C	22.949141	12.464140	0.958571
**C**	32.065707	23.302803	3.272841	C	17.444334	13.465298	1.980225
**C**	28.441016	18.951140	3.699318	C	16.551393	12.384907	2.216461
**C**	28.936884	17.620691	3.694297	C	17.086847	11.037864	2.349576
**C**	30.330697	17.389290	3.510912	C	18.465045	10.817365	2.096965
**C**	31.189665	18.481161	3.343026	C	23.835021	13.540957	0.700489
**C**	27.608772	14.105617	4.008241	C	20.332489	9.211599	1.832883
**C**	28.509302	15.189769	3.828442	C	22.082419	7.801999	1.477637
**C**	28.027925	16.527564	3.873424	C	16.853651	8.816160	2.823275
**C**	29.905506	14.939628	3.612557	C	18.931283	9.479825	2.172938
**C**	28.077172	12.753746	3.903532	C	16.944025	14.789494	1.941149
**C**	30.386195	13.602984	3.538257	C	15.548040	15.022850	2.047871
**C**	31.762311	13.385017	3.398437	C	14.656743	13.921298	2.189862
**C**	32.190258	15.743081	3.348744	C	15.183742	12.630325	2.308217

**C**	30.826434	16.021705	3.489323	C	23.431455	11.127306	0.910848
**C**	27.175287	11.661028	4.053913	C	24.763119	10.889222	0.583877
**C**	29.860109	11.168436	3.436880	C	25.653153	11.931882	0.310737
**C**	29.446940	12.493498	3.624027	C	25.196317	13.278662	0.391657
**C**	21.806949	26.370494	3.374185	C	20.015320	34.145560	0.103732
**C**	23.196380	26.121498	3.536904	C	27.080477	32.890045	0.821695
**C**	24.096979	27.204486	3.714931	C	34.138658	14.165256	3.153597
**C**	23.603605	28.536633	3.785955	C	29.440225	8.694303	3.286694
**C**	22.213639	28.785224	3.634023	C	9.966679	12.202777	2.453998
**C**	21.318660	27.702566	3.404486	C	7.522523	18.910971	1.365252
**C**	21.706540	30.123667	3.731179	C	27.643446	10.357069	3.856110
**C**	24.510845	29.620722	4.032046	C	24.158233	19.698678	0.033023
**C**	22.586957	31.200291	4.029087	C	24.627381	18.391386	0.164039
**C**	21.916504	32.499290	0.073753	C	26.148474	18.116977	4.070852
**C**	20.693279	32.746991	4.029523	C	26.647568	16.777942	4.090311
**C**	19.839505	31.688667	3.719350	C	25.616252	15.705285	0.209867
**C**	20.316057	30.384302	3.554410	C	26.095992	14.370533	0.168873
**C**	25.773722	29.392206	0.056157	N	32.941334	20.701995	2.964163
**C**	26.625963	30.460589	0.358429	N	33.366311	23.072875	3.026245
**C**	26.145228	31.758511	0.518054	N	11.275618	27.036626	1.773279
**C**	24.778566	31.988651	0.369064	N	10.843174	24.667571	1.773432
**C**	23.876653	30.957126	0.081096	N	15.355823	29.981990	2.206796
**C**	18.667818	20.709641	2.258409	N	13.118568	29.178737	1.819679
**C**	18.219289	22.018203	2.419642	N	32.438709	25.700915	3.537674
**C**	16.826583	22.278787	2.303777	N	30.601172	27.229199	3.844037
**C**	15.914031	21.203826	2.142293	N	20.797788	7.951274	1.817447
**C**	19.130930	23.113482	2.690988	N	22.974413	8.757914	1.175764
**C**	18.638281	24.446094	2.716695	N	16.280730	10.025792	2.719073
**C**	17.248914	24.699762	2.518641	N	18.118451	8.478110	2.549940
**C**	16.342060	23.618701	2.344748	H	22.587099	21.544177	3.502062
**C**	20.483553	22.897024	2.936640	H	28.536716	28.560721	4.049944
**C**	21.380486	23.936331	3.180058	H	32.259370	18.350399	3.191006
**C**	20.908286	25.277741	3.166479	H	22.577310	33.332576	0.315578
**C**	19.530328	25.527111	2.944241	H	18.771263	31.888135	3.620992
**C**	16.770346	26.031981	2.506026	H	19.735887	20.506963	2.307820
**C**	15.389508	26.279818	2.304602	H	20.856465	21.875356	2.937017
**C**	14.487429	25.208591	2.167867	H	10.692372	22.221941	1.789316
**C**	14.956579	23.871835	2.193882	H	17.650751	30.516503	2.897166
**C**	13.096585	25.476048	2.000716	H	22.030513	18.542570	0.820166
**C**	12.158328	24.416314	1.907424	H	20.320192	18.877248	1.520350
**C**	12.635829	23.040803	1.946829	H	14.544922	11.762728	2.462812
**C**	14.030687	22.791662	2.065863	H	25.077028	9.847237	0.561110
**C**	17.666949	27.125030	2.704856	H	19.196485	34.156467	0.838771
**C**	17.193156	28.464594	2.666085	H	19.744810	34.514816	3.252269

**C**	15.793195	28.718539	2.359068	H	20.799623	34.835738	0.441812
**C**	14.908384	27.619358	2.220748	H	27.581040	33.341004	3.987188
**C**	11.748945	21.969606	1.861488	H	27.956433	32.536218	1.382386
**C**	14.505835	21.456368	2.060991	H	26.575142	33.670772	1.407397
**C**	12.590434	26.799200	1.914732	H	34.456995	14.356088	2.116530
**C**	10.484110	25.959466	1.722987	H	34.373980	13.119566	3.394088
**C**	14.052923	30.130480	1.920894	H	34.733933	14.820710	3.805974
**C**	13.541066	27.914175	1.984060	H	29.238893	8.403596	2.243274
**C**	19.041088	26.874126	2.947404	H	28.913099	7.981723	3.936959
**C**	19.925276	27.957883	3.195723	H	30.520961	8.600046	3.460527
**C**	19.418676	29.288779	3.224324	H	10.454947	11.324112	2.896464
**C**	18.070343	29.512771	2.930319	H	9.571538	11.909000	1.468054
**C**	31.576753	24.671880	3.477469	H	9.110068	12.486494	3.082838
**C**	31.900296	26.909236	3.737735	H	6.935560	18.002834	1.557661
**C**	29.725841	26.209481	3.769156	H	7.339865	19.223075	0.325074
**C**	32.672313	14.435466	3.309680	H	7.153081	19.714440	2.019275
**C**	28.975182	10.097063	3.538200	H	23.093527	19.892497	0.148636
**C**	17.797243	19.641479	2.049652	H	32.145415	12.363842	3.375407
**C**	16.395477	19.875089	2.055559	H	32.903603	16.565681	3.277217
**C**	12.190069	20.641951	1.875161	H	30.901334	10.959271	3.187366
**C**	13.586919	20.376822	1.967936	H	9.387494	14.821687	2.184834
**C**	15.960867	17.453223	1.967436	H	12.666519	12.094675	2.423617
**C**	15.473311	18.786490	1.989634	H	9.496485	20.745395	1.546570
**C**	14.076327	19.031228	1.975262	H	8.766179	16.537687	1.674903
**C**	13.163649	17.940462	1.968283	H	26.950428	9.518019	3.935121
**C**	13.651719	16.607468	2.017238	H	22.451332	6.772520	1.443220
**C**	15.052471	16.364982	2.008252	H	16.207076	8.006206	3.174135
**C**	12.738964	15.503193	2.079781	H	9.412591	26.157734	1.625567
**C**	11.753523	18.190307	1.894244	H	13.711740	31.156047	1.751246
**C**	11.335591	15.738736	2.042608	H	34.768319	21.613227	2.614406
**C**	10.465487	14.650690	2.174833	H	32.608250	27.739431	3.820670
**C**	10.930751	13.343496	2.319466	H	27.693126	30.277182	0.493109
**C**	12.305053	13.118638	2.320307	H	24.409863	33.007575	0.494755

Table S2. H_2 evolution rate of N-CNS and TiO_2 P25 under visible light ($\lambda > 420$ nm).

**Photocatalyst**	**H_2 (µmol g^{-1} h^{-1})**	**Solution**
N-CNS	190	0.75 M Na_2S, 1.05 M Na_2SO_3/H_2O
TiO_2 P25	5.5	

Table S3. H_2 evolution rate of **N-CNS** and other materials reported in literature.

**Photocatalyst**	**H_2 (µmol g^{-1} h^{-1})**	**Solution**	**References**
BP/CN	427	Methanol/H_2O	S13
Procyanidin–methoxy-benzaldehyde (PC-MB)	252.02	H_2O	S14
Carbon-doped BN nanosheets	80	TEOA/H_2O	S15
Carbon dots/g-C_3N_4	27.6	TEOA/H_2O	S16
P-doped graphene	12	Methanol/H_2O	S17
N_2-COF	1.7	ACN/TEOA/H_2O	S18
Phenyl-triazine oligomers	121	pH 7, 0.5 M, phosphate buffer/TEOA/H_2O	S19
**N-CNS**	190	0.75 M Na_2S, 1.05 M Na_2SO_3/H_2O	**This work**

Supplementary References

1. Ramesh, E., Guntreddi, T., Sahoo, A. K. $AlCl_3$-catalyzed intermolecular annulation of thiol derivatives and alkynes by 1,2-sulfur migration: construction of 6-substituted benzo[b]thiophenes. *Eur. J. Org. Chem.* **2017**, 4405-4413 (2017).
2. Niu, L. T., Zhang, H., Yang, H. J., Fu, H. Metal-free iodination of arylboronic acids and the synthesis of biaryl derivatives. *Synlett* **25**, 995-1000 (2014).
3. Vo, T. H., Perera, U. G. E., Shekhirev, M., Pour, M. M., Kunkel, D. A., Lu, H. D., Gruverman, A., Sutter, E., Cotlet, M., Nykypanchuk, D., Zahl, P., Enders, A., Sinitskii, A. Sutter, P. Nitrogen-doping induced self-assembly of graphene nanoribbon-based two-dimensional and three-dimensional metamaterials. *Nano Lett.* **15**, 5770-5777 (2015).
4. Wang, J. Y., Zhu, Y. H., Zhuang, G. L., Wu, Y. Y., Wang, S. D., Huang, P. S., Sheng, G., Chen, M. Q., Yang, S. F., Greber, T., Du, P. W. Synthesis of a magnetic π-extended carbon nanosolenoid with Riemann surfaces. *Nat. Commun.* **13**, 1239 (2022).
5. Hisaki, I., Nakagawa, S., Tohnai, N., Miyata, M. A C_3-symmetric macrocycle-based, hydrogen-bonded, multiporous hexagonal network as a motif of porous molecular crystals. *Angew. Chem. Int. Ed.* **54**, 3008-3012 (2015).
6. Li, Y. S., Guo, L. S., Lv, Y. K., Zhao, Z. Q., Ma, Y. H., Chen, W. H., Xing, G. L., Jiang, D. L., Chen, L. Polymorphism of 2D imine covalent organic frameworks. *Angew. Chem. Int. Ed.* **60**, 5363-5369 (2021).
7. Wang, S. X., Tang, L., Cai, B. G., Yin, Z. M., Li, Y. F., Xiong, L., Kang, X., Xuan, J., Zhu, M. Z. Ligand modification of Au_{25} nanoclusters for near-infrared photocatalytic oxidative functionalization. *J. Am. Chem. Soc.* **144**, 3787-3792 (2022).
8. Zhang, S., Li, G., Li, L., Deng, X., Zhao, G., Cui, X., Tang, Z. Alloxan-catalyzed biomimetic oxidations with hydrogen peroxide or molecular oxygen. *ACS Catal.* **10**, 245 (2020).
9. Meng, Y., Luo, Y., Shi, J. L., Ding, H., Lang, X., Chen, W., Zheng, A., Sun, J., Wang, C. 2D and 3D porphyrinic covalent organic frameworks: The influence of dimensionality on functionality. *Angew. Chem. Int. Ed.* **59**, 3624 (2020).
10. Liang, X., Guo, Z., Wei, H., Liu, X., Lv, H., Xing, H. Selective photooxidation of sulfides mediated by singlet oxygen using visible-light-responsive coordination polymers. *Chem. Commun.* **54**, 13002 (2018).
11. Raza, F., Park, J. H., Lee, H. R., Kim, H. I., Jeon, S. J., Kim, J. H. Visible-light-driven oxidative coupling reactions of amines by photoactive WS_2 nanosheets. *ACS Catal.* **6**, 2754 (2016).
12. Zhou, P., Jiang, L., Wang, S., Hu, X., Wang, H., Yuan, Z., Zhang, Z. Synthesis of secondary aldimines from the hydrogenative cross-coupling of nitriles and amines over Al_2O_3-supported Ni Catalysts. *ACS Catal.* **9**, 8413 (2019).
13. Zhu, M. S., Kim, S., Mao, L., Fujitsuka, M., Zhang, J. Y., Wang, X. C., Majima, T. Metal-free photocatalyst for H_2 evolution in visible to near-infrared region:

Black phosphorus/graphitic carbon nitride. *J. Am. Chem. Soc.* **139**, 13234-13242 (2017).

14. Wu, Q. Y., Liu, Y., Cao, J. J., Sun. Y., Liao, F., Liu, Y., Huang, H., Shao, M. W., Kang, Z. H. A function-switchable metal-free photocatalyst for the efficient and selective production of hydrogen and hydrogen peroxide. *J. Mater. Chem. A* **8**, 11773-11780 (2020).
15. Huang, C. J., Chen, C., Zhang, M. W., Lin, L. H., Ye, X. X., Lin, S., Antonietti, M., Wang, X. C. Carbon-doped BN nanosheets for metal-free photoredox catalysis. *Nat. Commun.* **6**, 7698 (2015).
16. Qu, D., Liu, J., Miao, X., Han, M. M., Zhang, H. C., Cui, Z., Sun, S. R., Kang, Z. H., Fan, H. Y., Sun, Z. C. Peering into water splitting mechanism of g-C_3N_4-carbon dots metal-free photocatalyst. *Appl. Catal. B* **227**, 418-424 (2018).
17. Latorre-Sánchez, M., Primo, A., García, H. P-doped graphene obtained by pyrolysis of modified alginate as a photocatalyst for hydrogen generation from water-methanol Mixtures. *Angew. Chem. Int. Ed.* **52**, 11813-11816 (2013).
18. Banerjee, T., Haase, F., Savasci, G., Gottschling, K., Ochsenfeld, C., Lotsch, B. V. Single-site photocatalytic H_2 evolution from covalent organic frameworks with molecular cobaloxime Co-catalysts. *J. Am. Chem. Soc.* **139**, 16228-16234 (2017).
19. Schwinghammer, K., Hug, S., Mesch, M. B., Senker, J., Lotsch, B. V. Phenyl-triazine oligomers for light-driven hydrogen evolution. *Energy Environ. Sci.* **8**, 3345-3353 (2015).
